# Combination of ultrasound-based radiomics and deep learning with clinical data to predict response in breast cancer patients treated with neoadjuvant chemotherapy

**DOI:** 10.3389/fonc.2025.1525285

**Published:** 2025-06-05

**Authors:** Wu Tenghui, Liu Xinyi, Si Ziyi, Zhang Yanting, Ma Ziqian, Zhu Yiwen, Gan Ling

**Affiliations:** ^1^ Department of Nuclear Medicine, Xiangyang No. 1 People’s Hospital, Hubei University of Medicine, Xiangyang, China; ^2^ Department of Ultrasound, Xiangyang No.1 People’s Hospital, Hubei University of Medicine, Xiangyang, China; ^3^ Department of Oncology, The People’s Hospital of Zouping City, Zouping, China

**Keywords:** ultrasound, deep learning, breast cancer, neoadjuvant chemotherapy, radiomics

## Abstract

**Objectives:**

Accurate assessment of NAC efficacy is crucial for determining appropriate surgical strategies and guiding the extent of surgical resection in breast cancer. Therefore, this study aimed to design an integrated predictive model combining ultrasound imaging, deep learning features, and clinical characteristics to predict pCR in breast cancer patients undergoing NAC.

**Methods:**

A retrospective study was conducted, including 643 pathologically confirmed breast cancer patients who underwent NAC between January 2022 to February 2024 from two institutions (Center 1: 372 cases; Center 2: 271 cases). Ultrasound images before and after NAC were collected for each patient. A total of 2,920 radiomics features and 4,096 deep learning features were extracted from the ultrasound images. Multiple machine learning algorithms were employed to model and validate the diagnostic performance of different types of features. Finally, clinical data, radiomics, and deep learning features were integrated to form a fusion model, which was evaluated using receiver operating characteristic (ROC) analysis.

**Results:**

The combined model achieved the highest predictive performance for pathological complete response (pCR) across both cohorts. In the internal validation cohort, it reached an accuracy of 0.892 (95% CI: 0.862–0.912) and an AUC of 0.901 (95% CI: 0.854–0.948). In the external cohort, it maintained strong performance with an accuracy of 0.857 (95% CI: 0.822–0.928) and an AUC of 0.891 (95% CI: 0.848–0.934), significantly outperforming the individual models (DeLong test, p < 0.01).The deep learning model showed solid performance with accuracies of 0.875 and 0.833 in the internal and external cohorts, respectively, and AUCs of 0.870 and 0.874. The radiomics model displayed moderate accuracy and AUC in both cohorts, while the clinical model showed the lowest predictive capability among the models, with accuracy and AUC values around 0.67 in both cohorts.

**Conclusions:**

The combined model, integrating clinical, radiomics, and deep learning features, demonstrated superior predictive accuracy for pCR following neoadjuvant chemotherapy (NAC) in breast cancer patients, outperforming individual models. This integrated approach highlights the value of combining diverse data types to improve prediction, offering a promising tool for guiding NAC response assessment and personalized treatment planning.

## 1 Introduction

Breast cancer remains one of the most prevalent malignancies among women worldwide and is a leading cause of cancer-related mortality (1). Neoadjuvant chemotherapy (NAC) is widely employed as a first-line treatment strategy to downstage tumors before surgical intervention. Achieving a pathological complete response (pCR) following NAC is considered an effective surrogate endpoint for predicting long-term prognosis in breast cancer patients. Those who attain pCR have reported 5-year survival rates as high as 85–90% (2, 3).

Despite its benefits, the effectiveness of NAC varies significantly due to tumor heterogeneity, leading to considerable differences in pCR rates among different molecular subtypes of breast cancer, especially in advanced stages or in patients resistant to therapy (4–6). Approximately 30–50% of breast cancer patients achieve pCR after completing NAC, as defined by postoperative pathology (ypT0/is ypN0). Conversely, about 29% of patients exhibit no response to NAC, and 7.9% experience disease progression post-treatment, which adversely affects prognosis and increases mortality rates in advanced cases (2, 3). These disparities not only affect individual prognoses but also complicate treatment planning and decision-making processes. Consequently, there is a pressing need to understand the factors influencing NAC responsiveness and to develop reliable methods for predicting pCR in order to tailor individualized treatment strategies effectively.

The choice of surgical options after NAC largely depends on whether the patient achieves pCR. Some researchers suggest that patients who reach pCR may opt for breast-conserving surgery to improve quality of life and outcomes, with some even proposing the possibility of completely avoiding mastectomy. However, accurately identifying which patients are suitable for such conservative treatments remains a challenge in clinical practice. Currently, the assessment of NAC efficacy in clinical practice predominantly relies on subjective evaluations using ultrasound (US) and magnetic resonance imaging (MRI). While these imaging modalities provide valuable information, they have limitations in accurately predicting pCR due to factors like inter-observer variability and limited sensitivity and specificity. Moreover, the gold standard for determining pCR remains the pathological examination of surgical specimens obtained after NAC, which is invasive and only available postoperatively. This highlights a critical gap in preoperative assessment tools that can non-invasively and accurately predict NAC outcomes, enabling clinicians to optimize treatment plans before surgical intervention.

The emergence of deep learning and radiomics has opened new avenues for developing such predictive tools. Radiomics involves extracting a vast array of high-dimensional features from medical images, capturing subtle textural and spatial characteristics that are often imperceptible to traditional manual analysis (7). By modeling and integrating these features, robust predictive models can be established. Deep learning, with its powerful automated learning capability, effectively handles complex non-linear data relationships, further enhancing the model’s ability to capture abstract and spatial features, thereby improving the model’s predictive accuracy and robustness. With advancements in computational power and the accumulation of large-scale datasets, deep learning–based radiomics models have demonstrated substantial potential for clinical applications. The significance of this integration is that the two can play their relative advantages to describe different types of texture features, and finally achieve more accurate diagnostic efficiency through feature combination.

Several studies have demonstrated the feasibility of such approaches using other imaging modalities. For instance, Huang et al. (8) developed a predictive model using multimodal longitudinal MRI images for different pathological subtypes of breast cancer, achieving excellent diagnostic performance (AUC = 0.89), Song et al. also verified the feasibility of the method in prostate cancer (9). However, the reliance on MRI images across multiple time points limits the model’s applicability, given the higher cost, longer scanning time, and reduced accessibility of MRI compared to other imaging modalities.In clinical practice, ultrasound is the most commonly used and recommended modality for monitoring and evaluating NAC response in breast cancer due to its accessibility, cost-effectiveness, and real-time imaging capabilities. Despite these advantages, the quality of evidence for using ultrasound data to predict the efficacy of NAC in different pathological subtypes is still poor, and the method using radiomics combined with deep learning has not been explored (10). Therefore, the aim of this study is to develop a predictive model based on radiomics and deep learning using US images to predict patients’ pCR status. This model seeks to provide a non-invasive, practical tool that can assist clinicians in making more informed decisions regarding surgical planning and personalized treatment strategies.

## 2 Materials and methods

### 2.1 Patient information and clinical data

This retrospective study adhered to the Declaration of Helsinki and received ethical approval from Xiangyang First People’s Hospital and Zou Ping Hospital, with informed consent waived due to its retrospective nature. Data collection at Xiangyang First People’s Hospital involved 372 patients between January 2022 and February 2024, including 146 patients who achieved pathological complete response (pCR) and 226 patients who did not. These patients were divided into a training cohort and an internal validation cohort in a 7:3 ratio using stratified random sampling. Zou Ping Hospital collected data from 271 patients between March 2022 and February 2024, comprising 107 pCR and 164 non-pCR patients, which served as an external test cohort.

Inclusion criteria were as follows: (a) confirmed diagnosis of invasive breast cancer; (b) completion of NAC treatment followed by surgery; (c) availability of US data both before and at the midpoint of NAC; and (d) comprehensive clinical and pathological data. Although histological subtyping (e.g., ductal vs. lobular carcinoma) was not explicitly used as an inclusion criterion, the vast majority of patients were diagnosed with invasive ductal carcinoma.

Exclusion criteria included: (a) diagnosis of bilateral breast cancer; (b) incomplete or non-standardized NAC treatment or surgery; (c) poor US quality or absence of US data; and (d) presence of metastatic disease or a secondary malignancy.

**Figure 1:**
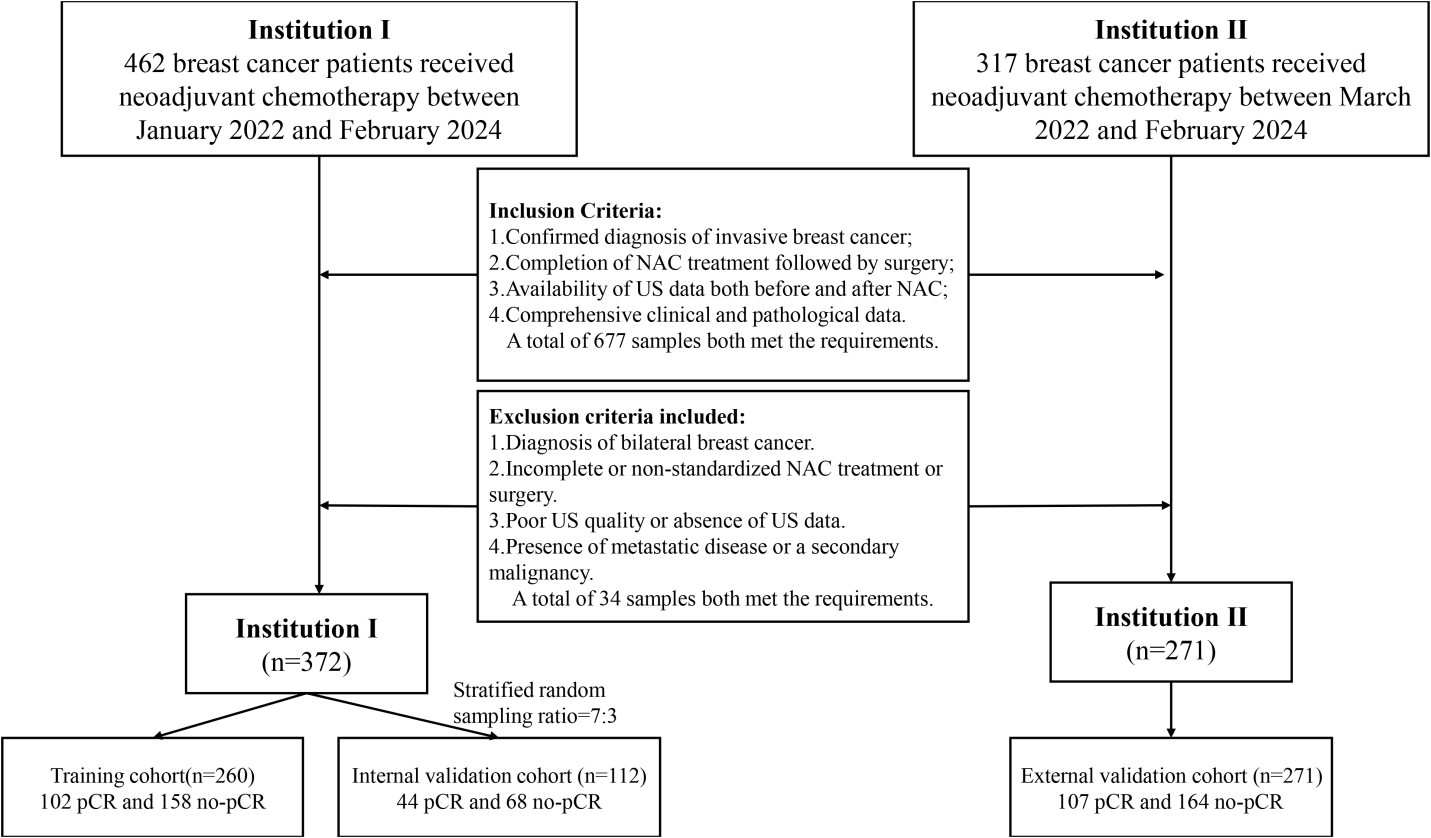
Diagram of the experimental inclusion-exclusion criteria.

Patient demographic data, including age and clinical symptoms, were obtained from medical records. Collected clinical data included: (a) age; (b) clinical stage; (c) estrogen receptor (ER) status; (d) progesterone receptor (PR) status; (e) HER-2 status; and (f) Ki-67 status. The comprehensive data screening and collection workflow is illustrated in **Figure 1**.

### 2.2 Experimental methods

#### 2.2.1 Data processing

Breast ultrasound examinations were conducted by five radiologists with over 10 years of experience in breast ultrasound imaging, both prior to intervention and at the midpoint of NAC. Imaging was performed using four different ultrasound systems (Resona 7, Mindray, China; Philips Healthcare, USA; LOGIQ E20, GE, USA; and Samsung, Korea), each equipped with a linear array transducer. To ensure consistency, all images were acquired at the largest cross-sectional area of the tumor.

To reduce variability introduced by different ultrasound machines, all images were rescaled to a uniform resolution of 512 × 512 pixels using linear interpolation. The 3-sigma method was applied to remove outlier pixel values. All segmentation was manually performed by two radiologists under the supervision of a senior breast imaging expert using 3D Slicer software. Radiologists were blinded to outcomes, and delineation was performed in consensus. The largest cross-sectional images from both pre-NAC and mid-NAC were used for segmentation and saved as ROI-original images.

To train the deep learning model, ROI-original images were further resampled to 128 × 128 pixels to generate a uniform dataset (ROI-resample) for input. This standardization ensured a consistent representation of tumor morphology and enhanced model generalizability.

#### 2.2.2 pCR prediction model

To construct the clinical model, clinical variables showing statistical significance (p < 0.05) in univariate analysis of the training cohort were selected and input into eight supervised machine learning algorithms. The radiomics model was developed by extracting features from the ROI-original images using the PyRadiomics library. Filters such as Laplacian of Gaussian and wavelets were applied to generate derivative images, from which 1,216 features per ROI were extracted. Categories included shape-based, first-order, GLCM, GLRLM, GLSZM, GLDM, and NGTDM features. Each patient contributed two ROIs (pre- and mid-NAC), resulting in 2,438 radiomics features per patient.

To construct the deep learning model, a ResNet-50 architecture was trained using the ROI-resample dataset. Probability-based predictions were generated through a softmax activation function in the final layer. The model with the best internal validation performance was selected. Deep features were extracted from the final fully connected layer for further integration.

To address class imbalance between pCR and non-pCR groups (~39% vs. ~61%), the Synthetic Minority Oversampling Technique (SMOTE) was applied to the training cohort. For algorithms that support class weighting, such as logistic regression and XGBoost, balanced class weights were also utilized.

**Figure 2:**
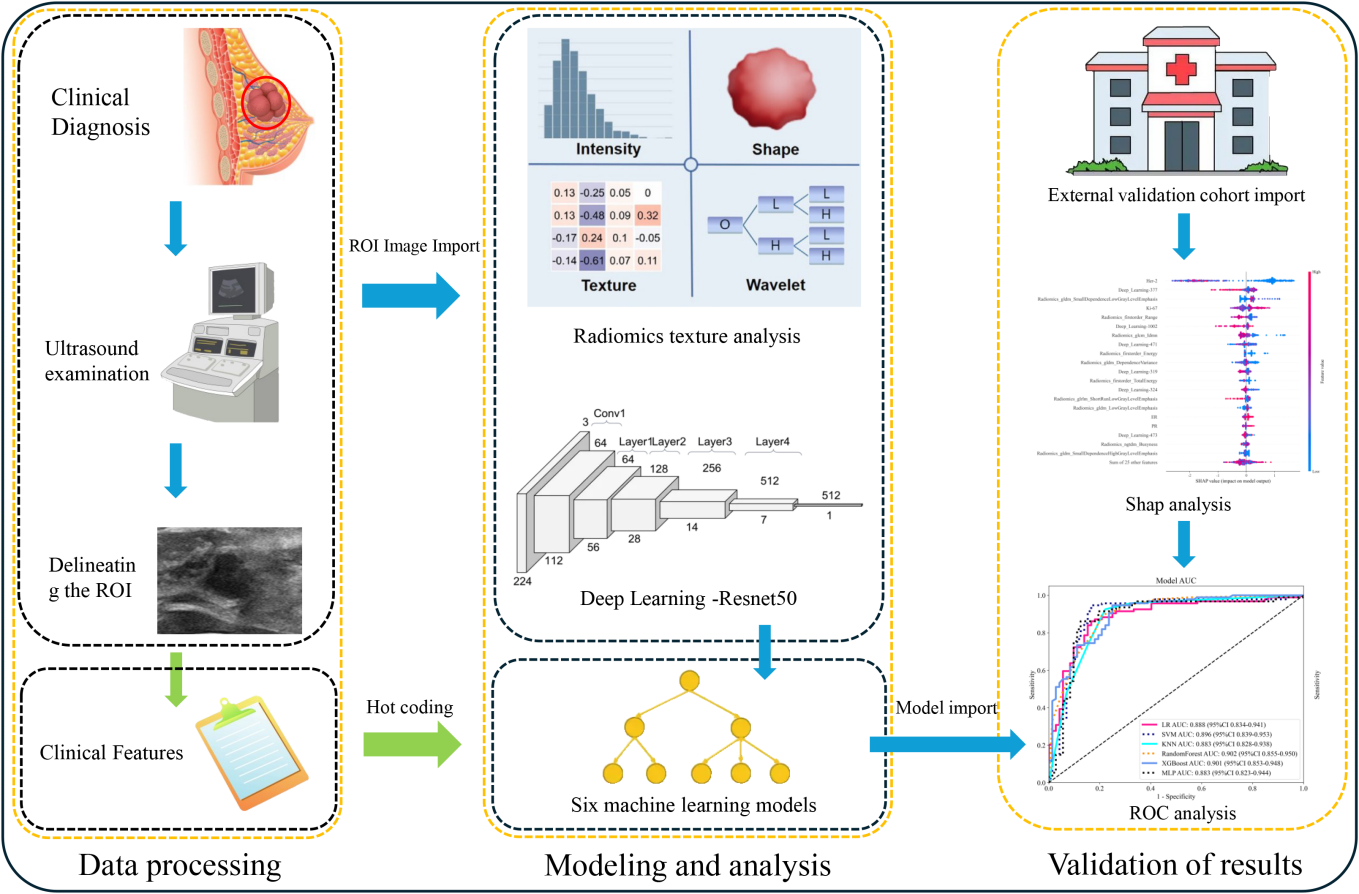
Flow chart. The flowchart shows the study design.

The composite (Combine) model was constructed by integrating clinical, radiomics, and deep learning features using early feature-level fusion. Eight machine learning algorithms were employed for model building. All models were trained on the training cohort and evaluated on both internal and external test sets. Model calibration was assessed using calibration curves and Brier scores. Clinical utility was evaluated using decision curve analysis (DCA). The complete experimental workflow is summarized in **Figure 2**.

#### 2.2.3 Statistical analysis

Statistical analysis and model construction were performed using R (version 4.1.3) and Python (version 3.6.2). For continuous variables, the Kolmogorov-Smirnov test was employed to assess normality. Depending on the distribution, either the t-test or the Mann-Whitney U test (using SciPy version 1.7.0) was used to compare differences between the two cohorts. For multivariate analysis, logistic regression was applied to evaluate associations between clinical variables and outcomes. The p-values were adjusted using the Benjamini-Hochberg correction to control for multiple comparisons, ensuring statistical rigor. Categorical variables were analyzed with the chi-square test to identify significant associations.

To assess the agreement between predicted probabilities and actual outcomes, model calibration was evaluated using calibration curves and Brier scores. Calibration curves were generated by plotting the predicted probabilities against observed event rates. The optimal classification threshold was determined based on the Youden index, maximizing the sum of sensitivity and specificity on the ROC curve. To further explore the clinical value of the model across different probability thresholds, DCA was performed, which estimates the net benefit of using the model in clinical decision-making compared to treating all or no patients.

To evaluate model performance, 95% confidence intervals (CIs) for the AUC were calculated using a bootstrapping approach with 1,000 iterations, providing robust interval estimates. Using the selected clinical features, a predictive model was developed using machine learning algorithms optimized for diagnostic accuracy.ROC curves were used to visually demonstrate the predictive ability of each model—the clinical, deep learning, radiomics, and composite models. Each model was tested on both internal and external validation sets to assess generalizability and predictive performance across different datasets. The DeLong test was conducted to compare the AUCs between models, allowing for statistical validation and comparison of their predictive capabilities.

## 3 Results

### 3.1 Baseline characteristics of patients

Between January 2022 and February 2024, a total of 372 patients were included in the primary cohort from Xiangyang First People’s Hospital, and 271 patients were included in the external validation cohort from Zou Ping Hospital (March 2022 to February 2024). In the primary cohort, 146 patients (39.2%) achieved pathological complete response (pCR), while 226 patients (60.8%) did not. Similarly, in the validation cohort, the pCR rate was 39.5% (107 out of 271), with the remaining 164 patients (60.5%) not achieving pCR.

**Table 1** summarizes the clinical characteristics of all patients in this study. The primary and validation cohorts exhibited similar baseline characteristics, with no significant differences observed in age or clinical stage between pCR and non-pCR patients across both cohorts (p = 0.631 and p = 0.682 in the primary cohort; p = 0.317 and p = 0.231 in the validation cohort, respectively). However, significant differences were noted in several molecular markers, including estrogen receptor (ER), progesterone receptor (PR), human epidermal growth factor receptor 2 (HER2), and Ki-67 status.

**Table 1:** Characteristics of patients in the training and test cohort.

Characteristics	Primary cohort (N=372)			Validation cohort 1 (N=271)		
	pCR (n=146)	N-pCR (n=226)	P Value	pCR (n=107)	N-pCR (n=164)	P Value
Age	48.93 ± 8.73	49.21 ± 9.66	0.631	47.91 ± 10.31	49.13.39 ± 9.78	0.317
Clinical Stage (%)			0.682			0.231
I	2 (1.37%)	1 (0.44%)		1 (0.9%)	2 (1.2%)	
II	97 (66.4%)	152 (67.3%)		67 (62.6%)	108 (65.9%)	
III	47 (32.2%)	73 (32.3%)		39 (36.4%)	54 (32.9%)	
ER Status (%)			<0.01			<0.01
Positive	65 (44.5%)	158 (69.9%)		63 (58.9%)	121 (73.8%)	
Negative	67 (45.9%)	82 (36.3%)		44 (41.1%)	43 (26.2%)	
PR status (%)			<0.01			<0.01
Positive	71 (48.6%)	159 (70.4%)		61 (57.0%)	122 (74.4%)	
Negative	68 (46.6%)	74 (32.7%)		46 (43.0%)	42 (25.6%)	
HER-2status (%)			<0.01			<0.01
Positive Negative	99 (67.8%) 33 (22.6%)	71 (31.4%) 169 (74.8%)		78 (72.9%) 29 (27.1%)	47 (28.7%) 117 (71.3%)	
Ki-67 Status (%)			0.036			0.01
Positive	106 (72.6%)	172 (76.1%)		86 (80.4%)	118 (72.0%)	
Negative	26 (17.8%)	68 (23.9%)		21 (19.6%)	46 (28.0%)	
Cancer subtype (%)			<0.01			<0.01
HR+/Her2-	17 (11.6%)	131 (58.0%)		13 (12.1%)	94 (57.3%)	
Her2+	97 (66.4%)	69 (30.5%)		78 (72.9%)	50 (30.5%)	
TN	21 (14.4%)	37 (16.4%)		16 (14.9%)	20 (12.2%)	

P-value is derived from the univariable association analyses between the clinicopathologic variables and Bone status. The data marked with * are averaged.

ER and PR positivity were more prevalent among non-pCR patients. In the primary cohort, ER positivity was observed in 69.9% of non-pCR patients compared to 44.5% in pCR patients (p < 0.01). Conversely, HER2 positivity was significantly higher in the pCR group, with rates of 67.8% in the primary cohort and 72.9% in the validation cohort (p < 0.01). Additionally, Ki-67 positivity, an indicator of cellular proliferation, was more common among pCR patients, showing significant differences in both cohorts (p = 0.036 in the primary cohort and p = 0.01 in the validation cohort).

Regarding molecular subtypes, the HER2-positive subtype had the highest pCR rate, with 66.4% of pCR patients in the primary cohort belonging to this subtype, whereas the hormone receptor-positive/HER2-negative (HR+/HER2−) subtype had the lowest pCR rate, accounting for only 11.6% of pCR patients (p < 0.01). These findings highlight significant associations between ER, PR, HER2, and Ki-67 status with pCR, underscoring the importance of these biomarkers in predicting NAC response.

### 3.2 Model performance

Achieved an accuracy (ACC) of 0.892 (95% CI: 0.862–0.912) and an area under the curve (AUC) of 0.901 (95% CI: 0.854–0.948). This model consistently outperformed the individual models. The deep learning model recorded an ACC of 0.875 (95% CI: 0.818–0.932) and an AUC of 0.870 (95% CI: 0.833–0.907), while the radiomics model had an ACC of 0.797 (95% CI: 0.791–0.913) and an AUC of 0.831 (95% CI: 0.788–0.873). The clinical model showed the lowest predictive capability, with an ACC of 0.674 (95% CI: 0.628–0.741) and an AUC of 0.682 (95% CI: 0.629–0.736).

In the external validation cohort, the combined model maintained high performance, achieving an ACC of 0.857 (95% CI: 0.822–0.928) and an AUC of 0.891 (95% CI: 0.848–0.934). The deep learning model demonstrated robust external generalizability with an ACC of 0.833 (95% CI: 0.791–0.875) and an AUC of 0.874 (95% CI: 0.838–0.909). The radiomics model also performed well, with an ACC of 0.801 (95% CI: 0.788–0.859) and an AUC of 0.822 (95% CI: 0.778–0.866). However, the clinical model recorded the lowest external validation performance, with an ACC of 0.655 (95% CI: 0.601–0.709) and an AUC of 0.666 (95% CI: 0.612–0.721).

**Figure 3:**
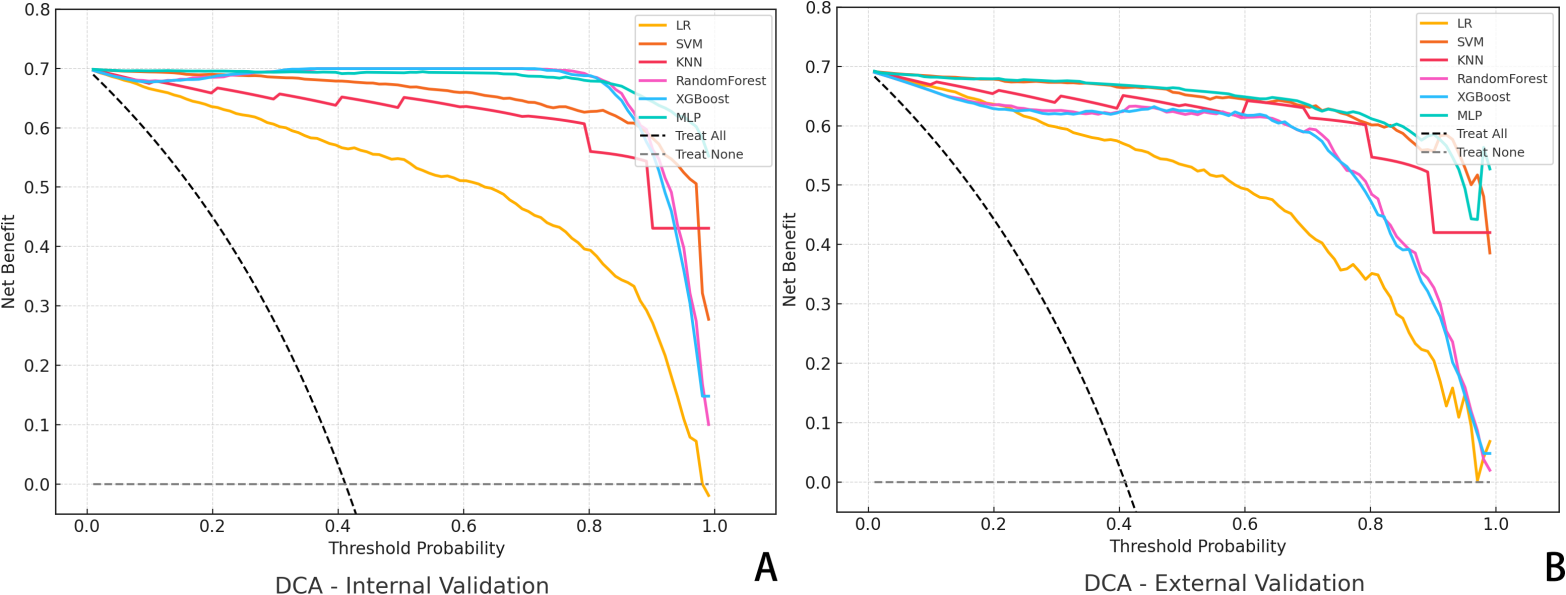
Decision Curve Analysis (DCA) for Predictive Models. **(A)** DCA curves of six algorithms in the internal validation cohort; **(B)** DCA results in the external validation cohort. The y-axis represents the net benefit, and the x-axis denotes the threshold probability.

**Figure 4:**
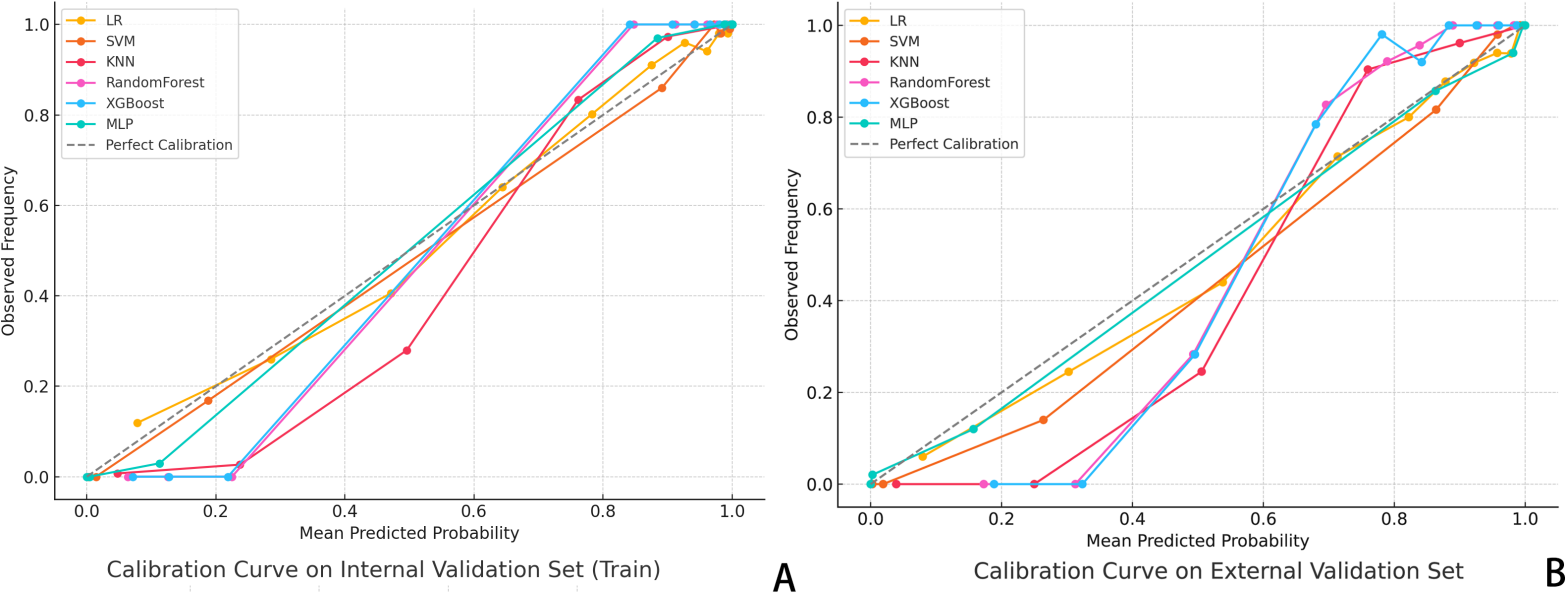
Calibration Curve Analysis. **(A)** Calibration curves for six classifiers in the internal validation set; **(B)** Dashed diagonal line indicates perfect calibration. A curve closer to the diagonal suggests better agreement between predicted probability and actual observed frequency of pCR. The XGBoost and MLP models showed the highest calibration accuracy across both datasets.

DCA was also conducted to assess the net clinical benefit across a range of threshold probabilities. The combined model provided the highest net benefit in both validation cohorts, supporting its clinical utility in decision-making contexts (**Figure 3**). To evaluate the reliability of probability-based predictions, calibration analysis was performed using calibration curves (**Figure 4**). The combined and deep learning models demonstrated good calibration performance, with curves closely aligned to the ideal diagonal and low Brier scores in both internal and external validation sets (see **Supplementary File S1**).

**Figure 5:**
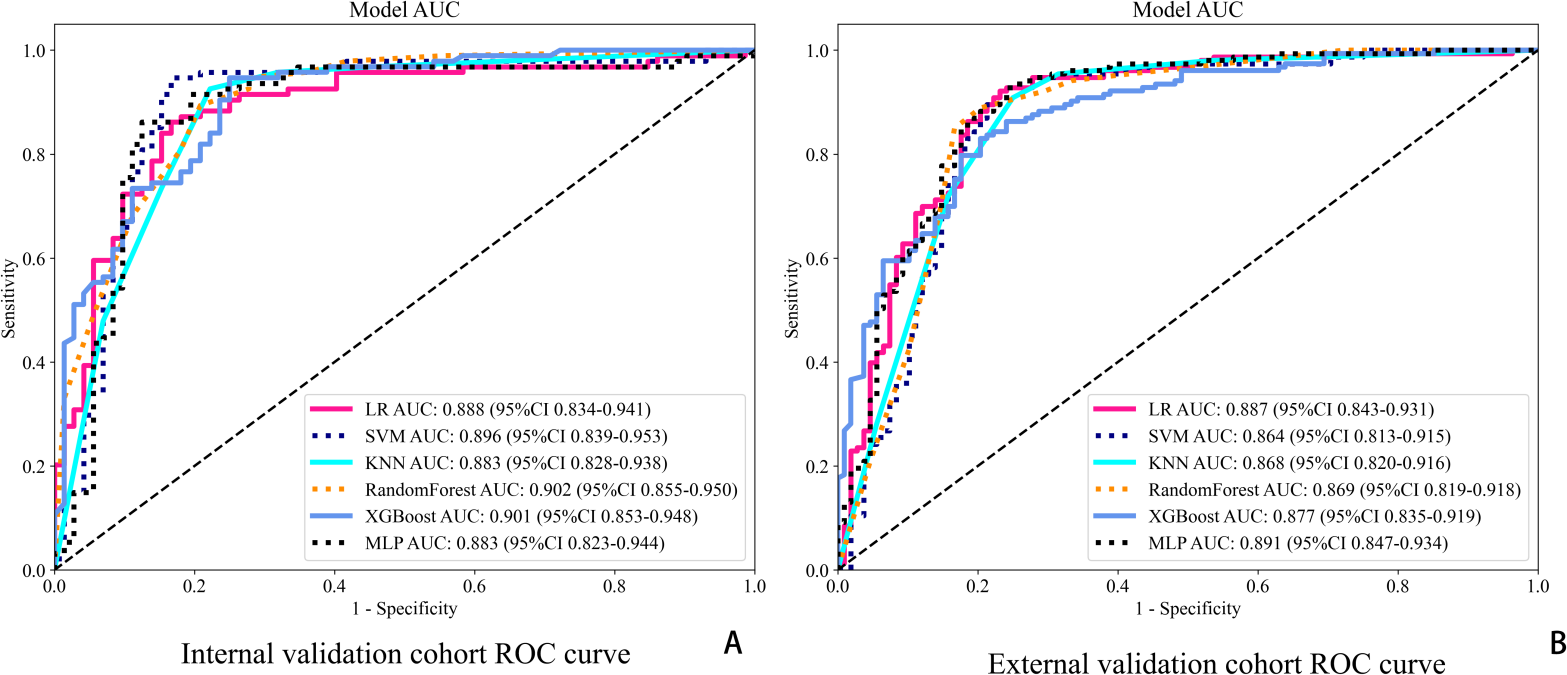
ROC curves for six classification models in both cohorts. **(A)** ROC curves in the internal validation cohort. **(B)** ROC curves in the external validation cohort.

For final model comparisons, DeLong’s test was used to compare the AUCs among the clinical, ResNet50, radiomics, and combined models. The results showed that the combined model significantly outperformed the clinical model (p < 0.01), and also demonstrated superiority over the radiomics and standalone deep learning models (see **Figure 5**, **Table 2**).

**Table 2:** Predictive model performance effectiveness.

Model	Accuracy		AUC		Delong test*	
	Internal validation cohort	External Validation	Internal validation cohort	External Validation	Internal validation cohort	External Validation
Combine	0.892 (0.862,0.912)	0.857 (0.822,0.928)	0.901 (0.854,0.948)	0.877 (0.834,0.919)	0.002	0.003
Deep learning	0.875 (0.818,0.932)	0.833 (0.791,0.875)	0.870 (0.833,0.907)	0.834 (0.808,0.889)	0.002	0.014
Radiomics	0.797 (0.791,0.913)	0.801 (0.788,0.859)	0.831 (0.788,0.873)	0.822 (0.778,0.866)	0.019	0.029
Clinic	0.674 (0.628,0.741)	0.655 (0.601,0.709)	0.682 (0.629,0.736)	0.666 (0.612,0.721)	–	–

1. DeLong test is performed with Clinic as the benchmark, and the 95% confidence interval is listed for AUC and ACC, respectively. 2. All results show the best model results in internal validation cohort AUC.

## 4 Discussion

Accurate prediction of pathological complete response (pCR) following neoadjuvant chemotherapy (NAC) is essential for optimizing surgical planning and improving outcomes for breast cancer patients. Reliable pCR prediction enables clinicians to make informed decisions about the feasibility of breast-conserving surgery, potentially avoiding unnecessary mastectomies and their associated morbidity (11–13). However, traditional reliance on imaging modalities like MRI and postoperative pathological examination presents limitations, including limited accessibility, high costs, and delayed diagnostic timing. Therefore, developing a convenient, non-invasive, and accurate method to assess pCR before surgery is a crucial objective in current breast cancer treatment strategies.

Recent advances in radiomics and deep learning have opened new avenues for enhancing the prediction of treatment response. Radiomics involves extracting high-dimensional quantitative features from medical images, capturing subtle textural, spatial, and morphological characteristics that may not be discernible through conventional imaging analyses (7). Deep learning, particularly convolutional neural networks (CNNs), can model complex non-linear relationships within imaging data, thereby improving predictive accuracy and robustness (14). Integrating these technologies with ultrasound imaging—a widely available, cost-effective, and non-invasive modality—offers a practical solution to overcome the limitations of traditional methods. Despite this potential, few studies have focused on using ultrasound-based radiomics models for predicting pCR in breast cancer.

In our study, we aimed to address this gap by developing a predictive model based on ultrasound images, leveraging the strengths of deep learning and radiomics to offer a practical and accessible tool for clinicians. Our model demonstrated strong performance in predicting pCR, with area under the curve (AUC) values of 0.907 and 0.862 across different validation cohorts. The use of ultrasound expands the applicability of predictive models to a broader patient population, including those in resource-limited environments or with contraindications to MRI. Beyond its accessibility, ultrasound’s real-time imaging capability enables dynamic monitoring of treatment response, further enhancing its clinical utility. By focusing on a widely available and user-friendly modality, our approach simplifies the predictive process, reduces methodological complexity, and facilitates more efficient clinical implementation. This not only improves workflow efficiency but also increases the likelihood of broader adoption in clinical practice, where ease of use is a critical factor for integrating new technologies.

In our cohort, the pCR rate following NAC was approximately 38%, which is slightly higher than the 26–35% typically reported in previous studies. This discrepancy may be attributed to the retrospective nature of our study, where patients were selected based on real-world clinical decisions. As a result, individuals with more favorable baseline characteristics—such as earlier-stage disease or molecular subtypes known to be more responsive to NAC—were more likely to be included. Moreover, the relatively limited sample size may have contributed to this deviation through statistical variability.Despite this potential selection bias, the reliability of our ultrasound-based radiomics model remains robust, as it leverages high-dimensional imaging features that are less influenced by subjective clinical judgment. This objectivity supports the model’s potential for broader clinical applicability and generalizability.

Currently, clinical evaluation of NAC response often includes biomarkers such as estrogen receptor (ER), progesterone receptor (PR), and human epidermal growth factor receptor 2 (HER2) status, which significantly influence treatment outcomes. Research has shown that molecular subtypes like triple-negative and HER2-positive breast cancers are more likely to achieve pCR compared to hormone receptor-positive tumors (15–20). Accordingly, our study incorporated these molecular subtypes as key clinical variables in the model development process. Univariate and multivariate logistic regression analyses revealed significant associations between molecular subtype, tumor grade, and the likelihood of achieving pCR (P < 0.05, **Table 1**), aligning with findings from previous research. However, predictive models based solely on traditional clinical indicators demonstrated limited accuracy (AUC of clinical feature model = 0.73, 0.69). This limitation could be due to the inherent complexity of tumor biology, where molecular and imaging markers alone may not fully capture the heterogeneity of treatment response. Additionally, certain clinical parameters, such as Ki-67 proliferation index and histological grade, may not always be reliably assessed due to sampling errors or variability in pathological interpretation (21, 22). These challenges underscore the necessity for advanced imaging-based models that integrate both clinical and imaging data to enhance predictive accuracy.

Radiomics research, leveraging high-throughput data and advancements in CNN-based deep learning, has significantly enhanced the non-invasive prediction of tumor biological behavior. Traditional radiomics approaches have demonstrated promise in identifying imaging features correlated with treatment outcomes, such as predicting pCR following NAC (10, 23, 24). However, our study introduces several methodological and clinical innovations that improve predictive accuracy and applicability beyond prior efforts.

Firstly, our model utilizes ultrasound imaging instead of MRI, which many existing models rely upon. Ultrasound offers substantial practical advantages due to its widespread availability, cost-effectiveness, and non-invasive nature, making it highly suitable for routine clinical practice. By integrating radiomics with deep learning, our model captures high-dimensional imaging features that are often undetectable through conventional analyses, enhancing the precision of pCR prediction in breast cancer patients undergoing NAC. The model’s strong performance metrics, with AUC values of 0.907 and 0.862 across different validation cohorts, underscore its effectiveness and potential for clinical application.

Secondly, we address the limitations of traditional radiomics models in capturing abstract and non-linear relationships within imaging data by incorporating CNNs. CNNs have the unique ability to extract complex spatial features from medical images through convolutional and pooling operations, analyzing relationships between distant pixels (25, 26). This capability provides deeper insights into tumor heterogeneity—a significant challenge in predicting treatment response in breast cancer. Furthermore, we enhanced the robustness and reliability of our predictive model through ensemble learning by combining radiomics and deep learning-derived features. In performing ensemble learning, we tested a variety of model structures, and the results showed that the XGBoost algorithm model had the highest accuracy in the internal validation set (**Supplementary File S1**). Employing the XGBoost algorithm, known for effectively handling non-linear and complex data interactions (27, 28), we developed the combined model. This ensemble model achieved impressive AUCs of 0.901 and 0.891 across two independent centers, demonstrating both its generalizability and clinical strength. The multicenter validation significantly enhances the external validity of our results. The DeLong test (P < 0.05) revealed significant differences between our ultrasound-based model and traditional clinical risk models, emphasizing the necessity of incorporating advanced imaging techniques into predictive modeling.

**Figure 6:**
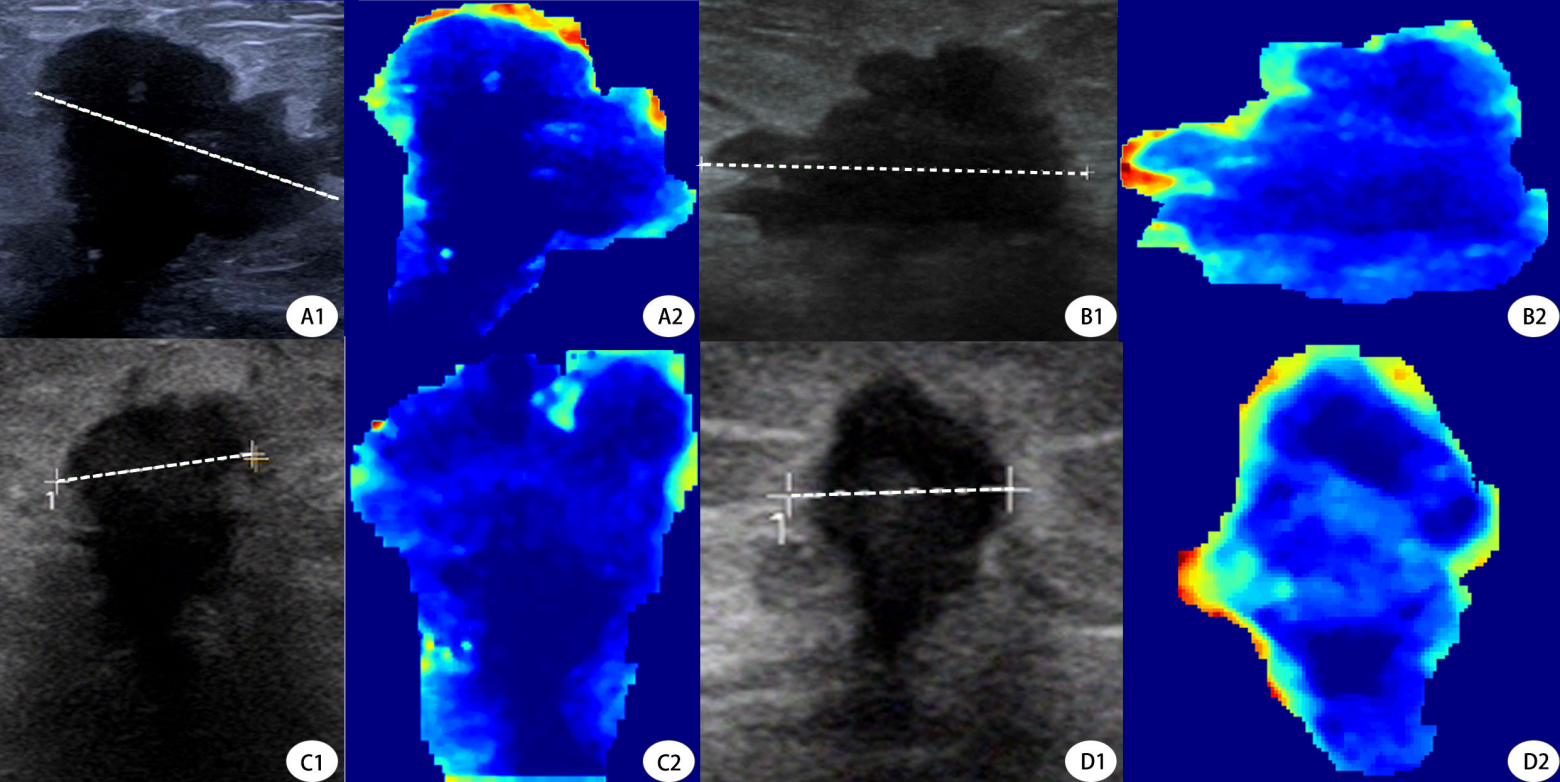
Grad-CAM Visualization of Deep Learning Model Attention in Pre- and Post-NAC Ultrasound Images. This figure demonstrates the deep learning model’s attention maps using Gradient-weighted Class Activation Mapping (Grad-CAM) on tumor ultrasound images before and after neoadjuvant chemotherapy (NAC). (A1, A2) Pre- and post-NAC ultrasound and Grad-CAM images, respectively, of a 53-year-old patient who did not achieve pCR. The Grad-CAM heatmap (A2) highlights strong peripheral activations, particularly on the upper tumor border.B1, B2 Corresponding post-NAC ultrasound and Grad-CAM images of the same non-pCR patient. The attention remains at the edge but appears more diffuse, indicating persistent residual tumor.C1, C2 Pre-NAC ultrasound and Grad-CAM visualization of a 49-year-old patient who achieved pCR. The heatmap (C2) shows dispersed and weak activations across the tumor, suggesting limited model attention toward aggressive patterns.(D1, D2) Post-NAC ultrasound and Grad-CAM of the same pCR patient. The model’s attention in D2 is minimal and centrally located, aligning with radiologic signs of tumor regression.Dashed lines represent the maximal tumor diameters measured during routine clinical evaluation. Tumor sizes were A = 2.24 cm, B = 2.61 cm (non-pCR case), and C = 1.08 cm, D = 0.68 cm (pCR case), respectively. These measurements further validate model attention correlates with tumor shrinkage patterns.

**Figure 7:**
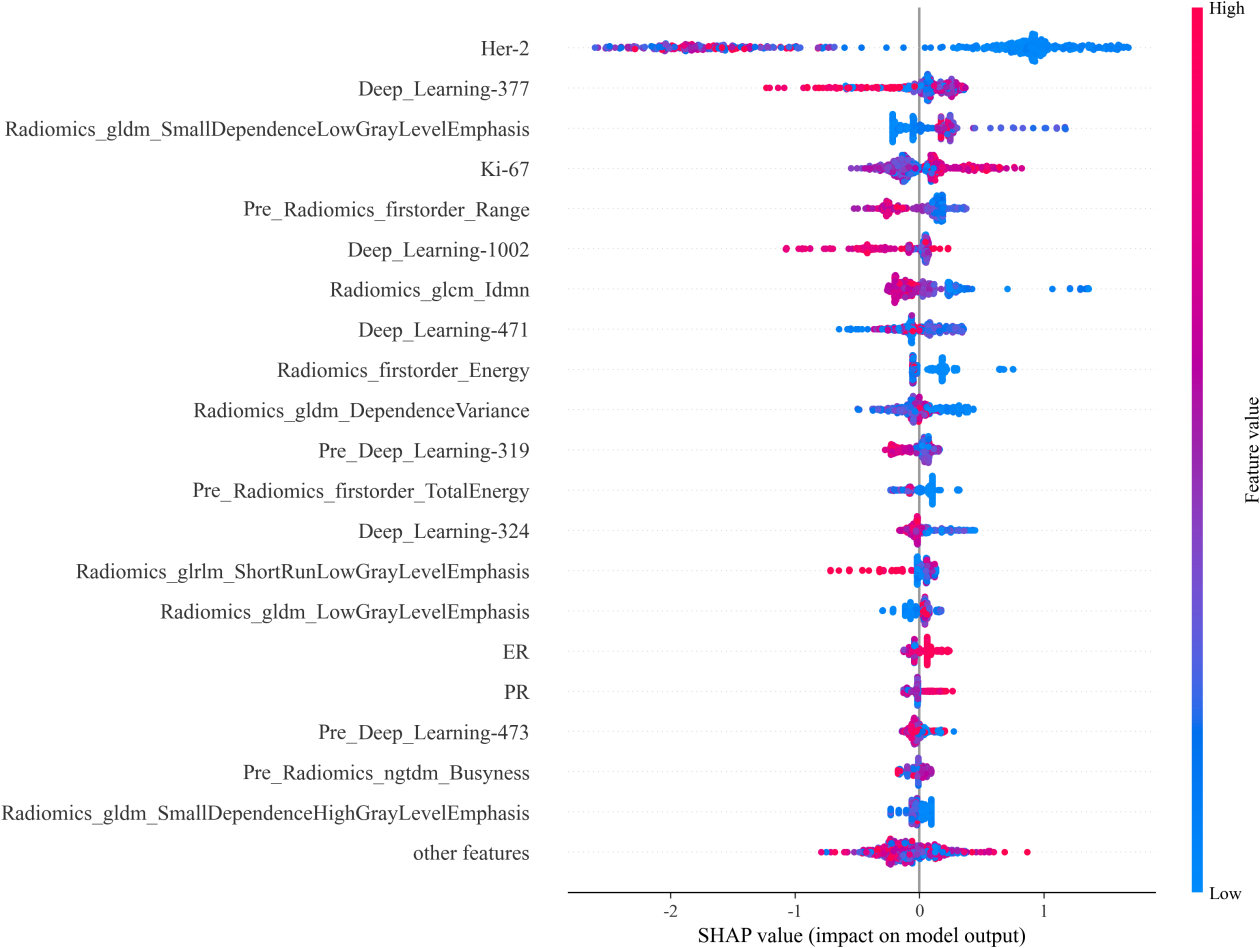
SHAP Plot. The SHAP values illustrate each feature’s contribution to the prediction outcome, providing insight into feature importance and the model’s interpretability.

To address the “black box” nature of deep learning, we incorporated Gradient-weighted Class Activation Mapping (Grad-CAM) into our study to visually interpret which tumor regions the model prioritized for prediction (**Figure 6**). This technique generates heatmaps that indicate the areas most influential in the model’s decision-making. For interpretability, we further utilized SHAP plots (**Figure 7**) to reveal the decision-making process of the optimal model (internal validation set). As a novel visualization tool, SHAP showed that among the top 20 features, clinical features were all considered and prioritized by the model, with molecular phenotypes like Ki-67 and HER2 positively correlated with pCR probability, consistent with previous research. Notably, both deep learning and radiomic features were among the top 20, validating our integrated learning strategy that combines different types of features. Importantly, clinical reviewers who evaluated the SHAP plots confirmed not only the intuitive alignment of high-impact variables—such as HER2 and Ki-67—with their clinical expectations, but also found the explanations actionable in supporting individualized treatment discussions. Feedback indicated that the clear ranking and directionality of feature contributions could help reinforce clinical decision-making, particularly when used alongside other interpretable tools like Grad-CAM.

In addition to evaluating discriminative performance via AUC, we assessed the calibration of our model’s predicted probabilities. Calibration curves showed a strong agreement between predicted and actual pCR probabilities across both internal and external cohorts, suggesting that the model not only distinguishes outcomes effectively but also provides reliable probability estimates. Furthermore, DCA demonstrated that our integrated model yielded a greater net clinical benefit across a wide range of threshold probabilities, compared with models based solely on clinical or radiomic features. These findings underscore the practical value of our approach, indicating that the model’s high discriminative power is matched by strong calibration and tangible clinical utility.

In addition to visual interpretability, we conducted calibration curve analyses (**Figure 4**) and decision curve analysis (DCA, **Figure 7**) to further evaluate the clinical reliability and practical value of our model. Calibration curves demonstrated that the predicted probabilities aligned well with the actual outcomes in both internal and external validation sets, as reflected by Brier scores of 0.102 and 0.109, respectively, supporting the model’s reliability. Moreover, DCA showed that the combined model consistently provided the highest net benefit across a wide range of threshold probabilities, confirming its strong clinical utility in decision-making scenarios. Notably, the optimal probability threshold for distinguishing pCR from non-pCR cases may vary depending on the clinical context. In our analysis, threshold values between 0.4 and 0.7 offered the best balance between sensitivity and specificity, as observed from DCA performance, and corresponded to the range where net clinical benefit was maximized across most patient scenarios. This range was selected based on maximizing clinical utility while maintaining interpretability for real-world application.

Our model demonstrated excellent performance in predicting pCR, providing a more accessible and cost-effective alternative to MRI-based models. This is particularly important in routine clinical settings where resource constraints or contraindications to MRI may limit its use. Additionally, the use of ultrasound imaging has the potential to expand the applicability of predictive models to a broader patient population, including those in resource-limited settings. Beyond its accessibility, ultrasound enables clinicians to monitor treatment response dynamically, further enhancing its clinical utility.

Furthermore, our model demonstrates not only excellent performance in terms of discrimination and calibration but also delivers consistent net benefit and clinical interpretability through robust visual explanation tools. This positions the model as a promising, scalable, and user-friendly solution for preoperative pCR prediction, particularly in resource-limited or MRI-constrained clinical environments. By focusing on ultrasound—a widely accessible and low-cost modality, we further simplify model deployment and enhance feasibility for routine integration into clinical workflows.

Despite the promising results of our study, several limitations should be acknowledged. First, the model relies on manual tumor segmentation for feature extraction, which introduces potential variability due to operator dependency. In this study, all segmentations were performed in consensus by two experienced radiologists to mitigate inter-observer variability. However, we acknowledge that inter-observer agreement was not formally quantified, which may impact reproducibility. This limitation has been noted, and future studies will incorporate quantitative evaluation of segmentation consistency using standard metrics such as the Dice Similarity Coefficient. Moreover, although manual delineation remains common in radiomics research, the development of automatic or semi-automatic segmentation methods will be critical to improve reproducibility, reduce labor, and enhance clinical applicability. We plan to explore these approaches in subsequent work.

Second, while our use of ultrasound imaging offers practical advantages such as accessibility and cost-effectiveness, ultrasound is inherently operator-dependent, and variations in image acquisition and quality could affect radiomic feature extraction and model performance. This variability underscores the importance of standardizing ultrasound scanning protocols and ensuring adequate training across institutions to promote consistency and reduce noise in future multicenter implementations. Notably, in our preprocessing, we implemented uniform image resampling and pixel normalization strategies to reduce inter-equipment variability, thus improving the consistency of feature extraction.

Third, although our cohort included only patients with confirmed invasive breast cancer who underwent NAC, we did not further stratify cases based on molecular subtypes such as hormone receptor (HR) or HER2 status. Certain subtypes—such as HER2-negative Luminal A—are less responsive to NAC and are often not recommended for such treatment. However, since this was a retrospective study, all patients had already received NAC based on clinical judgment and established treatment guidelines. This real-world selection process likely excluded low-response subtypes and reduced potential molecular-level bias. This is supported by our data: while HR+/HER2− patients accounted for approximately 38.5% of the total cohort, they represented only 11.6% of the pCR group, consistent with their known lower chemosensitivity. In contrast, HER2-positive and triple-negative breast cancer (TNBC) patients accounted for 46.8% and 15.0% of the cohort, respectively, and demonstrated significantly higher pCR rates, in alignment with existing clinical evidence. These distributions are highly consistent with real-world NAC-treated populations, suggesting that our cohort is representative and clinically relevant. Nevertheless, future studies should consider incorporating molecular subtyping more explicitly into model development to further improve performance across heterogeneous tumor biology.

Finally, while our model demonstrated strong predictive performance across two independent centers, the relatively limited sample size and geographic diversity may restrict its generalizability to broader populations. To address this, we plan to expand our external validation to include geographically and ethnically diverse cohorts across multiple clinical centers. Preliminary collaborations have already been initiated with two additional tertiary hospitals outside our current regional network, and ethics approval processes are underway. This will ensure the robustness and scalability of our model in real-world applications. Additionally, future research should continue to explore strategies that enhance interpretability—such as explainable artificial intelligence (XAI)—and develop intuitive clinical decision support tools that facilitate seamless integration into clinical workflows.

## 5 Conclusions

In this study, we developed a deep learning-based radiomics model using ultrasound imaging to predict pCR in breast cancer patients undergoing NAC. Integrating CNN allowed for the extraction of complex, non-linear imaging features, addressing the limitations of traditional radiomics approaches in capturing tumor heterogeneity. By employing ultrasound, we ensured that our model is both accessible and cost-effective, making it suitable for widespread clinical application. Additionally, ensemble learning, through the combination of radiomics and deep learning-derived features, further enhanced the predictive accuracy and robustness of the model. The multicenter validation demonstrated strong generalizability across independent datasets, confirming the potential of our model in clinical practice.

## Data Availability

The raw data supporting the conclusions of this article will be made available by the authors, without undue reservation.

## References

[1] Sung H Ferlay J Siegel RL Laversanne M Soerjomataram I Jemal A. Global cancer statistics 2020: GLOBOCAN estimates of incidence and mortality worldwide for 36 cancers in 185 countries. CA: A Cancer J Clin. (2021) 71:209–49. doi: 10.3322/caac.21660 33538338

[2] Harbeck N Nitz UA Christgen M Kümmel S Braun M Schumacher C. De-escalated neoadjuvant trastuzumab-emtansine with or without endocrine therapy versus trastuzumab with endocrine therapy in HR+/HER2+ Early breast cancer: 5-year survival in the WSG-ADAPT-TP trial. J Clin Oncol: Off J Am Soc Clin Oncol. (2023) 41:3796–804. doi: 10.1200/JCO.22.01816 36809046

[3] Göker E Hendriks MP van Tilburg M Barcaru A Mittempergher L van Egmond A. Treatment response and 5-year distant metastasis-free survival outcome in breast cancer patients after the use of MammaPrint and BluePrint to guide preoperative systemic treatment decisions. Eur J Cancer (Oxford England: 1990). (2022) 167:92–102. doi: 10.1016/j.ejca.2022.03.003 35421703

[4] de Nonneville A Houvenaeghel G Cohen M Sabiani L Bannier M Viret F. Pathological complete response rate and disease-free survival after neoadjuvant chemotherapy in patients with HER2-low and HER2–0 breast cancers. Eur J Cancer (Oxford England: 1990). (2022) 176:181–8. doi: 10.1016/j.ejca.2022.09.017 36257173

[5] Chen JH Bahri S Mehta RS Carpenter PM McLaren CE Chen WP. Impact of factors affecting the residual tumor size diagnosed by MRI following neoadjuvant chemotherapy in comparison to pathology. J Surg Oncol. (2014) 109:158–67. doi: 10.1002/jso.23470 PMC4005994 24166728

[6] Wolf DM Yau C Wulfkuhle J Brown-Swigart L Gallagher RI Lee PRE. Redefining breast cancer subtypes to guide treatment prioritization and maximize response: Predictive biomarkers across 10 cancer therapies. Cancer Cell. (2022) 40:609–623.e6. doi: 10.1016/j.ccell.2022.05.005 35623341 PMC9426306

[7] Lambin P Rios-Velazquez E Leijenaar R Carvalho S van Stiphout RG Granton P. Radiomics: extracting more information from medical images using advanced feature analysis. Eur J Cancer (Oxford England: 1990). (2012) 48:441–6. doi: 10.1016/j.ejca.2011.11.036 PMC4533986 22257792

[8] Huang Y Zhu T Zhang X Li W Zheng X Cheng M. Longitudinal MRI-based fusion novel model predicts pathological complete response in breast cancer treated with neoadjuvant chemotherapy: a multicenter, retrospective study. EClinicalMedicine. (2023) 58:101899. doi: 10.1016/j.eclinm.2023.101899 37007742 PMC10050775

[9] Song X Zhang S Shi T Huang X Wang Y Du M. A machine learning radiomics model based on bpMRI to predict bone metastasis in newly diagnosed prostate cancer patients. Magnetic Resonance Imaging. (2024) 107:15–23. doi: 10.1016/j.mri.2023.12.009 38181835

[10] Li Z Liu X Gao Y Lu X Lei J. Ultrasound-based radiomics for early predicting response to neoadjuvant chemotherapy in patients with breast cancer: a systematic review with meta-analysis. Radiol Med. (2024) 129:934–44. doi: 10.1007/s11547-024-01783-1 38630147

[11] Jannusch K Dietzel F Bruckmann NM Morawitz J Boschheidgen M Minko P. Prediction of therapy response of breast cancer patients with machine learning based on clinical data and imaging data derived from breast [18F]FDG-PET/MRI. Eur J Nucl Med Mol Imaging. (2024) 51:1451–61. doi: 10.1007/s00259-023-06513-9 PMC10957677 38133687

[12] Sella T Simor B Adler-Levy Y Maly B Kadouri L Carmon E. MRI prediction of neoadjuvant chemotherapy response is equivalent in patients with or without mammographic calcifications: a step towards adapting surgical approach? Eur Radiol. (2023) 33:7168–77. doi: 10.1007/s00330-023-09640-x 37086288

[13] Zhou J Lu J Gao C Zeng J Zhou C Lai X. Predicting the response to neoadjuvant chemotherapy for breast cancer: wavelet transforming radiomics in MRI. BMC Cancer. (2020) 20:100. doi: 10.1186/s12885-020-6523-2 32024483 PMC7003343

[14] Dayarathna S Islam KT Uribe S Yang G Hayat M Chen Z. Deep learning based synthesis of MRI, CT and PET: Review and analysis. Med Image Anal. (2024) 92:103046. doi: 10.1016/j.media.2023.103046 38052145

[15] Wu HY Lin CY Tzeng YD Hung CC Liu SI Yin CH. Preoperative systemic inflammation response index: Clinicopathologic predictor of pathological complete response in HER2-positive breast cancer patients receiving neoadjuvant systemic therapy. J Chin Med Assoc. (2024) 87:226–35. doi: 10.1097/JCMA.0000000000001034 PMC12718766 38095571

[16] Curigliano G Burstein HJ Gnant M Loibl S Cameron D Regan MM. Understanding breast cancer complexity to improve patient outcomes: The St Gallen International Consensus Conference for the Primary Therapy of Individuals with Early Breast Cancer 2023. Ann Oncol. (2023) 34:970–86. doi: 10.1016/j.annonc.2023.08.017 37683978

[17] Loibl S Poortmans P Morrow M Denkert C Curigliano G. Breast cancer [published correction appears in Lancet. Lancet. (2021) 397:1750–69. doi: 10.1016/S0140-6736(20)32381-3 33812473

[18] Encinas G Maistro S Pasini FS Katayama ML Brentani MM Bock GH. Ki-67 index after neoadjuvant endocrine therapy as a prognostic biomarker in patients with ER-positive/HER2-negative early breast cancer: a systematic review and meta-analysis. Eur J Cancer. (2023) 194:113358. doi: 10.1016/j.ejca.2023.113358 37857118

[19] Hong R Xu B. Breast cancer: an up-to-date review and future perspectives. Cancer Commun (Lond). (2022) 42:913–36. doi: 10.1002/cac2.12358 PMC9558690 36074908

[20] van den Ende NS Nguyen AH Jager A Kok M Debets R van Deurzen CHM. Triple-negative breast cancer and predictive markers of response to neoadjuvant chemotherapy: A systematic review. Int J Mol Sci. (2023) 24:2969. doi: 10.3390/ijms24032969 36769287 PMC9918290

[21] Gown AM. The biomarker ki-67: promise, potential, and problems in breast cancer. Appl Immunohistochem Mol Morphol. (2023) 31:478–84. doi: 10.1097/PAI.0000000000001087 36730064

[22] Whelan TJ Smith S Parpia S Fyles AW Bane A Liu FF. Omitting radiotherapy after breast-conserving surgery in luminal A breast cancer. N Engl J Med. (2023) 389:612–9. doi: 10.1056/NEJMoa2302344 37585627

[23] Zhu T Huang YH Li W Zhang YM Lin YY Cheng MY. Multifactor artificial intelligence model assists axillary lymph node surgery in breast cancer after neoadjuvant chemotherapy: multicenter retrospective cohort study. Int J Surg. (2023) 109:3383–94. doi: 10.1097/JS9.0000000000000621 PMC10651262 37830943

[24] Jiang W Deng X Zhu T Fang J Li J. ABVS-based radiomics for early predicting the efficacy of neoadjuvant chemotherapy in patients with breast cancers. Breast Cancer (Dove Med Press). (2023) 15:625–36. doi: 10.2147/BCTT.S418376 PMC10439736 37600669

[25] Lenharo M. An AI revolution is brewing in medicine. What will it look like? Nature. (2023) 622:686–8. doi: 10.1038/d41586-023-03302-0

[26] Wang H Pujos-Guillot E Comte B de Miranda JL Spiwok V Chorbev I. Deep learning in systems medicine. Brief Bioinform. (2021) 22:1543–59. doi: 10.1093/bib/bbaa237 PMC8382976 33197934

[27] Fan Z Jiang J Xiao C Chen Y Xia Q Wang J. Construction and validation of prognostic models in critically Ill patients with sepsis-associated acute kidney injury: interpretable machine learning approach. J Transl Med. (2023) 21:406. doi: 10.1186/s12967-023-04205-4 37349774 PMC10286378

[28] Zheng J Zhang Z Wang J Zhao R Liu S Yang G. Metabolic syndrome prediction model using Bayesian optimization and XGBoost based on traditional Chinese medicine features. Heliyon. (2023) 9:e22727. doi: 10.1016/j.heliyon.2023.e22727 38125549 PMC10730568

